# Ingestible electronic sensors to measure instantaneous medication adherence: A narrative review

**DOI:** 10.1177/20552076221083119

**Published:** 2022-02-28

**Authors:** Peter R Chai, Clint Vaz, Georgia R Goodman, Hannah Albrechta, Henwei Huang, Rochelle K Rosen, Edward W Boyer, Kenneth H Mayer, Conall O’Cleirigh

**Affiliations:** 1 Department of Emergency Medicine, 1861 Brigham and Women’s Hospital, Boston, MA, USA; 2 446213 The Fenway Institute, Fenway Health, Boston, MA, USA; 3 Department of Psychosocial Oncology and Palliative Care, Dana Farber Cancer Institute, Boston, MA, USA; 4 The Koch Institute for Integrated Cancer Research, Massachusetts Institute of Technology, Cambridge, MA, USA; 5 Department of Psychiatry, 2348 Massachusetts General Hospital, Boston, MA, USA; 6 The Centers for Behavioral and Preventive Medicine, 23324 The Miriam Hospital, Providence, RI, USA; 7 Department of Behavioral and Social Sciences, 174610 Brown University School of Public Health, Providence, RI, USA; 8 Division of Infectious Diseases, Beth Israel Deaconess Medical Center, Boston, MA, USA

**Keywords:** Ingestible sensor, ingestible pill, digital pill, medication adherence, electronic adherence, digital medicine

## Abstract

**Objective:**

Medication nonadherence contributes to significant morbidity and mortality worldwide. While many techniques to measure adherence exist, digital pill systems represent a novel, direct method of measuring adherence and a means of providing instantaneous adherence supports. In this narrative review, we discuss digital pill system research based on clinical trials and qualitative investigations conducted to date and potential future applications of digital pill system in medication adherence measurement.

**Methods:**

We conducted a literature search in PubMed of English language peer-reviewed articles describing the use of digital pill system for medication adherence measurement between 2000 and 2021. We included all articles that described the deployment of ingestible sensors and those involving qualitative investigations of digital pill system with human subjects.

**Results:**

A total of 95 articles were found on initial search; 75 were removed based on exclusion criteria. Included articles were categorized as investigations that deployed an ingestible sensor in human populations (*n* = 18), or those that conducted qualitative work (*n* = 3). For pilot studies, the mean accuracy of the sensor to successfully detect a medication ingestion event ranged from 68% to 100%. When digital pill systems were deployed in real-world clinical settings, accuracy ranged from 68% to 90% with lower accuracy due to nonadherence to digital pill system technology. Qualitative studies demonstrated that providers and patients perceive the digital pill system as a facilitator for improving adherence and as a potential platform for delivering adherence interventions. Additionally, ingestion data from digital pill system was viewed as useful in facilitating adherence discussions between clinicians and patients.

**Conclusions:**

This narrative review demonstrates that the use of digital pill system is broadly feasible across multiple disease states including human immunodeficiency virus, hepatitis C infection, solid organ transplants, tuberculosis, schizophrenia, cardiovascular disease, and acute fractures, where adherence is closely linked to significant morbidity and mortality. It also highlights key areas of research that are still needed prior to broad-scale clinical deployment of such systems.

## Introduction

Maintaining adherence to pharmacotherapy is difficult to achieve. Many barriers to adherence exist, ranging from experiencing or anticipating drug-related adverse events, to the economic and social burden of acquiring medications, to the social stigmas associated with certain medications and disease states.^1^ It is estimated that at least 50% of individuals who are on medications for chronic disease management are nonadherent to their prescribed regimens.^2^ The cost of nonadherence is significant; nonadherence to medication accounts for between $100 and $290 billion, or 13%, of total healthcare costs in the United States (US) annually.^3,4^ There is wide variation around the cost of adherence depending on disease state. A meta-analysis from 2018 calculated the all-cause annual cost per person for medication nonadherence to be between $5271 and $52,341.^5^ In the US, 69% of hospital admissions due to medication-related events are a result of medication nonadherence.^6^

Multiple methods for measuring ingestion events exist. These include indirect measures which are surrogate techniques that infer the presence of medication ingestions, as well as direct measures, which have the capacity to conclusively confirm ingestion events.^7^ Both direct and indirect techniques have advantages and disadvantages; indirect measures of adherence, like pharmacy refill data or individual self-report, are easy to operationalize but may provide incomplete and inaccurate data. Electronic adherence monitoring systems like smart pill bottles which record and transmit data on when the pill bottle is opened provide a technological solution to adherence, but this technique is indirect and can overestimate adherence.^8^ Direct measures like directly observed therapy (DOT) and video observed therapy (VOT) provide verification of medication ingestion events but may have decreased acceptance in individuals with privacy concerns or stigmatized conditions and may be costly to implement.^9^ Pharmacologic measurement of adherence, where the drug or metabolite is measured in the human matrix, is also direct measures but may require specialized laboratory support making them currently infeasible for broad adoption.

With increasing adoption of digital health tools in the management of chronic diseases, many other devices and systems have emerged as methods for increasing medication adherence.^10–12^ These include the use of standalone robotic systems that provide “nudges” or behavioral prompts programmed around expected ingestion times to smart medicine cabinets to better measure access and ingestion of medications.^13^ The ultimate goal of these digital health interventions is to provide increasingly reliable data around medication adherence in an unobtrusive manner. Combined with machine learning techniques and aggregated individualized adherence data, predictions on adherence and nonadherence may enable antecedent reminders around adherence in specific situations.^14^ The majority of these systems have undergone some pilot testing but have yet to be deployed and demonstrated as efficacious in powered clinical trials.^15,16^

One novel method for directly measuring medication adherence is a digital pill system (DPS).^17^ DPSs are composed of a radiofrequency emitter integrated into a standard gelatin capsule, which creates a digital pill. The digital pill then over encapsulates a desired medication via manual placement of the drug inside the capsule, or via a standard pill filling machine. Once the digital pill is ingested, exposure to gastric chloride ions energizes the radiofrequency emitter, which projects a signal outside the body that is acquired by a wearable Reader device. The Reader acts as a relay, storing and forwarding ingestion data to a cloud-based server that can display instantaneous, on-demand adherence data to users and key stakeholders, including health care providers and research teams. The radiofrequency emitter is coated in a food grade epoxy to prevent leaching of metals into the body, transits the gastrointestinal tract and is excreted in feces. There are no absolute contraindications to use of the DPS outside of known hypersensitivity reactions to the metal components of the radiofrequency sensor (silver and magnesium). To date, two DPSs have been cleared by the US Food and Drug Administration (FDA) for the measurement of medication ingestion events.^18,19^ One (Proteus Digital Health) utilizes a cutaneous patch adherent to the abdominal wall to collect ingestion data.^20^ The other (etectRx) utilizes an off-body wearable device for the same purpose.^21^

## Objective

In this narrative review, we discuss the current state of DPS technology for the direct measurement of medication adherence, describe the methods in which it has been deployed to date across various disease states, and suggest potential future applications of DPS in the context of medication adherence measurement.

## Methods

We conducted a literature search in PubMed of English language, peer-reviewed manuscripts describing the use of DPS for medication adherence measurement between 2000 and 2021. The search term included the following keywords: (“ingestible sensor” OR “digital pill” OR “ingestible sensor”) AND (“medication adherence” OR “electronic adherence” OR “digital medicine”). Manuscripts were downloaded and abstracts were reviewed by a member of the study team, who distilled each article into several key points. Because we sought to perform a narrative review on the clinical use of DPS for medication adherence monitoring, we only included manuscripts that described the deployment of these sensors and qualitative investigations in clinical human subjects. We therefore excluded manuscripts that exclusively described engineering advances, pharmacokinetics, case reports, and reviews of previous literature. Preprints, non-peer-reviewed concept pieces, and letters to the editor were also excluded.

We grouped articles into two categories: those that primarily reported on a clinical trial conducted with human subjects, and those that utilized qualitative methods to explore concepts related to DPS. For the clinical trials, we recorded the number of participants, type of DPS used, study design, disease state investigated, and primary and secondary outcomes. For the qualitative investigations, we identified the specific behavioral or technology theories that were used to ground interview or focus group guides, the type of qualitative techniques used, and important themes discovered.

All articles were collated and discussed by two members of the study team (CV, PRC) (Table 1). In the event of discrepancies in interpretation of the articles reviewed, a third study team member evaluated the article and rendered a decision on categorization. The results were presented and discussed among all members of the study team.

**Table 1.** Characteristics of included studies.

Authors	Study design	Population	Main findings
Au-Yeung et al.^22^	Pilot demonstration project	*n* = 111 healthy volunteers and patients with tuberculosis, heart failure, and hypertension.	The positive and negative detection accuracy of the system when compared to directly observed ingestion was 97.1% and 97.7%, respectively.
Belknap et al.^24^	Pilot demonstration project	*n* = 30 tuberculosis patients who are enrolled for DOTS co-ingested the sensor with their ATT medication.	The positive detection accuracy of the ingestible sensor system was 98.1% when the three percent data missed because of prototype malfunction and when a repeat measure model was used it increased to 99.6%. The identification accuracy was 100%.
Bonacini et al.^10^	Pilot demonstration project	*n* = 28 patients with chronic HCV infection treated on fixed dose combination of ledipasvir/sofosbuvir.	89% of the patients had medication adherence >/ = 95% during the study period. 26 (93%) of the study participants attained sustained virological response when assessed after 12 weeks or more of posttreatment. Of the two unresponsive cases one had suboptimal adherence (<90%) and other was due to drug resistance.
Chai et al.^25^	Pilot demonstration project	*n = *15 opioid-naïve individuals who were discharged from the emergency department with acute fracture pain.	The study was able to detect the actual patterns of opioid ingestion and found that most of the opioid naive patients with acute fracture only use opioids during initial days depending on their perception of their pain.
Chai et al.^26^	Pilot demonstration project	*n* = 10 patients with acute extremity fractures.	The DPS was able to detect the ingestion events with 87% accuracy. The decrease in accuracy is because of two participants who failed to charge the batteries of the system due to acute pain. The satisfaction survey among patients following the DPS use revealed that the DPS is palatable, easy to use, and valuable.
Daar et al.^27^	Pilot demonstration project	*n* = 15 PLWH on stable antiretroviral therapy (ARV) with suboptimal adherence (<90%) over last 28 days self-reported or found by clinician on 6 months of clinical evaluation)	More than 70% of the participants stated that they tolerated the system well and it is comfortable to use. Over 80% agreed that the system is helpful.
DiCarlo et al.^28^	Pilot demonstration project	*n* = 37 hypertensive patients.	The wearable sensor detection of 510 valsartan doses was 98%. Mean taking and timing adherence rates between clinical visits were 90% and 83%, respectively.
Eisenberger et al.^29^	Pilot demonstration project	*n* = 20 stable adult kidney transplants.	Out of 4136 ingestible event markers, enteric coated mycophenolate sodium capsules (IEM-ECMPS) prescribed 2824 (68%) ingestions have been detected without being directly observed. 100% of 34 directly observed ingestion events were detected accurately. The detection rate of daily medication ingestion was 99.3% in patients who took two IEM-ECMPS capsules BID and 100% in those who took one IEM-ECMPS capsule twice daily.
Flores et al.^30^	Pilot demonstration project	*n* = 20 healthy volunteers.	100% of directly observed ingestion events are detected. Of 384 self- administered ingestion events 371 got detected accurately (96.6%). Of the 391 recorded ingestion events 385 (98.47%) were remotely transmitted from the reader to a secure cloud storage. The overall adherence to the prescribed study capsules as measured by the DPS was 97.75%.
Frias et al.^31^	Cluster-randomized pilot clinical trial	*n* = 120 participants with uncontrolled hypertension and diabetes mellitus.	The participants who used the DPS had a significant reduction of −21.8 mmHg (SE 2.5) in systolic blood pressure (BP) when compared to −12.7% (SE 2.8) for the usual care group at 4 weeks. 81% from the digital medicine group achieved their BP goal of 140/90 mmHg, whereas only 33.3% among the usual care group achieved the BP goal. The patients who used the DPS had greater reductions in HbA1c, DBP, and LDL-C.
Kamal et al.^32^	Pilot demonstration project	*n* = 15 people living with HIV (PLWH) and *n* = 6 health care providers (HCPs) who are directly involved in their health care.	Eight of the 15 PLWH used the digital medicine were supportive, and the rest found it inconvenient. HCPs commented that the text message reminder in the study is the most important aspect, and they would recommend it to people with adherence difficulties for short-term use, up to 6 months.
Moorhead et al.^33^	Post-hoc analysis	Data of *n* = 57 patients participated in a cluster-randomized clinical trial among patients with uncontrolled hypertension and type 2 diabetes gathered in this efficacy study and *n* = 74 patients in the safety study.	This analysis suggested that the digital health medication dose reminder in patients with low medical adherence was the most important function of DPS and there was no incidence of overdosing due to medication dose time reminder during the study period.
Noble et al.^34^	Pilot demonstration project	*n * = 39 hypertensive patients and *n* = 15 practicing pharmacists.	The mean change in SBP in patients who used the DPS over the 2-week evaluation period was − 7.9 ± 22.1 mmHg; mean change in DBP was − 2.8 ± 12.9 mmHg. 32% of the study participants have uncontrolled hypertension due to poor medication adherence. 15 patients participated in the post intervention survey and all of them found the system experience was positive. 91% of the pharmacists surveyed agreed that the DPS helped them in assessing the actual medication ingestion trends and giving their patients specific recommendations.
Peters-Strickland et al.^35^	Pilot demonstration project	*n* = 67 adult patients with schizophrenia and their health care providers.	78% of patients rated were somewhat satisfied, satisfied, or extremely satisfied with the DPS and 70% of them found it somewhat helpful, helpful, or extremely helpful. 65% of them rated it as somewhat easy, easy, or very easy to use the system.
Profit et al.^36^	Pilot demonstration project	*n* = 30 and *n* = 29 healthy volunteers for two sub studies.	The overall accuracy of ingestion detection was 78.3% during sub study A. The overall accuracy of ingestion detection in sub study B is 96.6%. The mean latency of time from ingestion signal detection by wearable sensor to detection by cloud-based server is 6.2–10.3 min. 50% of the ingestions detected before 2 min and 90% of the detection occurred within 30 min of ingestion.
Sulkowski et al.^37^	Pilot demonstration study	*n* = 288 adults with chronic HCV infection.	Sustained virological response was achieved in 99.1% of 218 participants who had HCV RNA assessed at ≥ 10 weeks post-treatment.
Thompson et al.^38^	Pilot demonstration project	*n* = 28 patients with high CVD risk attending cardiac prevention and rehabilitation.	90% of the study participants self-reported that they have good adherence (never missed a medication) but when adherence was directly measured using the DPS only 57% found to have good adherence (> 80%).
Triplett et al.^39^	Pilot demonstration project	*n * = 33 pediatric solid organ transplant patients. 21 of them participated in the satisfaction survey. Five health care providers involved directly in the transplant completed the satisfaction survey.	27 participants discontinued the study prior to 6 months. The main reason behind withdrawal from the study was the inconvenience with the patch used in the system. Even though, majority of the study participants felt that DPS increases motivation and sense of control.
Chai et al.^40^	Qualitative study	n = 30 participants of age >18 years, self-reported HIV negative, cis-gender MSM, currently on pre-exposure prophylaxis (PrEP) or eligible for PrEP, and self-reported substance abuse excluding alcohol with in last six months.	Participants described that the DPS could be helpful during events of substance abuse and times of spontaneous sexual behavior, when there is a high chance of failure to adhere to medication. *n* = 19 participants showed willingness to use the DPS for monitoring real-time preexposure prophylaxis adherence. While few had concerns of safety, size of the reader, stigma and some due to lack of knowledge about the system.
Holender et al.^41^	Focus group discussion	*n* = 12 cardiovascular patients aged >65 years participated in two focus groups.	The participants suggested that the DPS is welcoming once it's familiar and they feel that this could improve medication monitoring by the health care providers, prevent adverse events, reduce confusion with polypharmacy and prolong independence. The barriers discussed were lack of cognitive abilities and memory problems in elderly and difficulty in familiarizing new technology.
de Mendoza et al.^42^	Qualitative study	*n* = 10 healthcare providers treating breast cancer survivors participated in qualitative semi structured interviews. *n* = 19 participated in an online survey.	Providers consider the side effects, access, forgetfulness, knowledge and beliefs, and communication are the barriers for the patients to adhere to hormonal therapy and many of their participants have adherence issues. They were confident that DPS can provide them timely and accurate adherence information and also help their patients to adhere to the medication. They think that privacy, safety, and access would be the patient's concerns with DPS.

## Results

**Figure 1.**
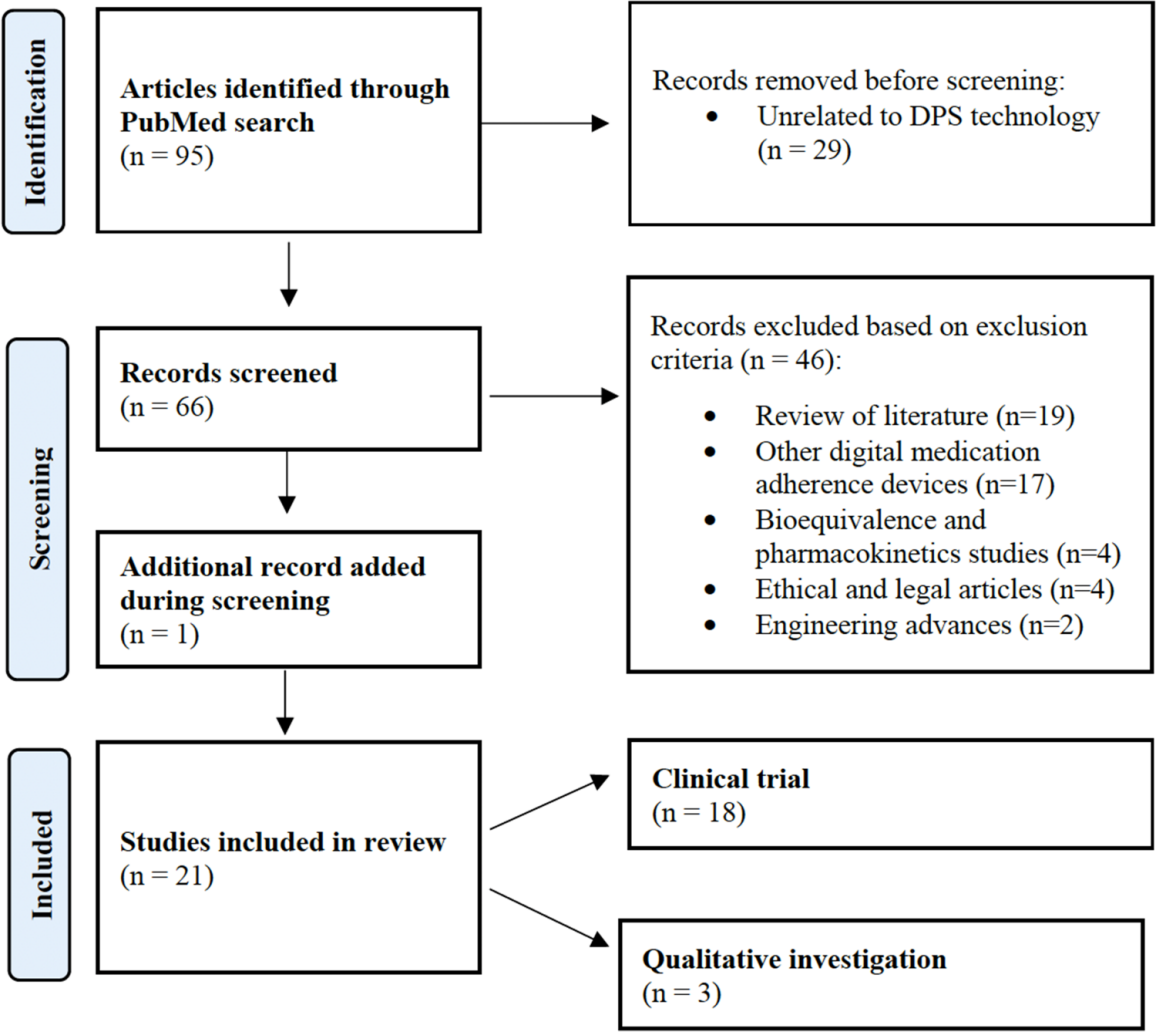
Article selection process ***.*** Note: Review conducted per PRISMA 2020 guidelines for systematic reviews.^23^

During the period from 2000 to 2021, our PubMed search yielded 95 manuscripts (Figure 1). Of these, 75 articles were eliminated based on the exclusion criteria. Most manuscripts excluded were reviews of previous literature (*n*  =  19) and manuscripts surrounding other digital technologies for medication adherence (*n*  =  17). The remaining 20 articles were accessed and included for review. We also discovered one additional article which was not covered in our initial search terms, but referenced in several other manuscripts.^22^ After discussion, because the content of this article related to the deployment of digital pills in human subjects, we decided to include it in our analysis. Therefore, a total of 21 manuscripts were evaluated, including 18 clinical trials that deployed an ingestible sensor in human populations, and three investigations involving exploratory qualitative work (Table 1). No manuscripts contained a discussion of cost effectiveness or an economic analysis of DPS in selected disease states.

### Human clinical trials involving use of a DPS

Of the 18 clinical trials that reported on the deployment of a DPS in human subjects, most were single-arm pilot studies (*n*  =  16).^22,24–30,32,34–39,43^ Two were cluster-randomized pilot clinical trials among individuals with uncontrolled hypertension and diabetes.^31,33^ All 18 investigations deployed the DPS with a primary endpoint of demonstrating the feasibility of their use in select patient populations where medication adherence is important. Disease states studied included human immunodeficiency virus (HIV), hepatitis C infection, solid organ transplants, tuberculosis, schizophrenia, cardiovascular disease (CVD), and acute fractures. The duration of all included pilot studies, ranged from 1 week to 6 months. In all clinical trials, participants were asked to ingest a digital pill over-encapsulating the study drug except for one investigation where the DPS was formulated separately from the study drug. In this single investigation, the digital pill acted as an indirect measure of adherence.^24^

In the pilot studies, the mean accuracy of successful detections of medication ingestion events via the radiofrequency sensor ranged from 68% to 100%.^22,24,26,29,30,36^ The methods for determining the ground truth of total actual ingestions varied across investigations. Some investigators utilized the number of original pills dispensed as the number of expected ingestions; others used participant self-report or pill counts of unused pills as the ground truth.^25,26^ Detection accuracy, as assessed in four pilot investigations, ranged from 97.1% to 100% in the setting of directly observed digital pill ingestions. When the DPS was deployed in the real world, detection accuracy ranged from 68% to 90%.^22,24,29,30^ Most of the reduction in detection accuracy in real-world studies was due to nonadherence to the DPS technology or failure of the technology. Three investigations showed that a DPS can detect simultaneous ingestions (i.e. ingestion of two different digital pills at the same time); to accomplish this, unique radiofrequency signatures were pre-programmed into the digital pills, permitting the wearable receiver to acquire two different signals despite their proximity to each other.^22,26,29^ These investigations demonstrated a 99.6% positive detection rate in the context of simultaneous ingestions.^22,29^

While most of the pilot studies reviewed sought to define the feasibility of using a DPS in an indicated disease state, four focused on biological outcomes.^31,34,37,43^ Two pilot investigations were conducted among individuals with hepatitis C, who used the DPS with either ledipasvir/sofosbuvir or glecaprevir/pibrentasvir or fosbuvir/velpatasvir. In the first study, all participants had preexisting risk factors for medication non-adherence. After a median of 13 weeks, their sustained virological response was 99.1% (*n*  =  216); at this point, participants had used the DPS for a median of 92.9% of their prescribed treatment time. The median rate of adherence to the DPS was 95%.^37^ The primary outcome in the second study was the percentage of participants with greater than or equal to 95% medication ingestion adherence; a reported 89% (*n*  =  25) of participants achieved this benchmark. As a secondary outcome, the researchers also considered sustained virological response (SVR) and cure at 8 or 12 weeks, depending on each participant's treatment plan. Participants that achieved SVR also had a higher adherence rate as reported by the DPS.^43^ In the third study that evaluated physiological outcomes, investigators deployed the DPS among individuals with uncontrolled hypertension to provide evidence-based blood pressure (BP) recommendations. Key findings included a mean change in systolic BP of − 7.9  ±  22.1 mmHg and diastolic BP of − 2.8  ±  12.9 mmHg over a two-week period.^34^ Finally, a fourth study utilized a cluster-randomized pilot clinical trial design. Individuals who had failed treatment with two or more antihypertensives with systolic BP >140 and type 2 diabetes with glycated hemoglobin level >7% were randomized to use the DPS for 4 or 12 weeks, as compared to standard use of antihypertensives without a DPS. The primary endpoint was to determine if the use of an ingestible sensor improved BP and diabetes mellitus control. Participants who used the DPS had a significant reduction of −21.8 mmHg (SD 2.5) in systolic BP when compared to −12.7% (SD 2.8) for the standard care group for 4 weeks. A total of 81% from the DPS group achieved their BP goal of 140/90 mmHg, while 33.3% in the standard care group achieved their BP goal.^31^

The 18 clinical trials involved the use of two different DPS. The Proteus Digital Health Feedback System (DHFS) was deployed in 16 studies, in which participants used a cutaneous receiver patch to acquire the ingestion signal from the digital pill.^22,24,27–39,43^ The etectRx ID-Cap system was deployed in two studies and involved the use of a body-worn receiver.^25,26^ Of the 16 investigations that utilized the DHFS, 10 studies reported local skin irritation, described as inflammation, at the patch site in 115 of 872 (13.2%) participants.^22,24,27–29,31,35,38,39,43^ Additionally, a total of 27 (3.09%) individuals dropped out of these investigations due to challenges adhering to the wearable patch.^27,29,35,39^ Twenty of these dropouts were pediatric organ transplant patients who had enrolled in a study to measure adherence to post-transplant medications.^39^

All investigations obtained ethical clearance from an institutional review board before starting the study, except one study that deployed a DPS for adherence measurement among individuals with hypertension. The authors of this investigation reported that the study was determined to be a minimal risk because adherence data were de-identified and no alterations in the standard of care in hypertensive treatment occurred among participants; therefore, no ethics board approval was obtained in this case.^34^

Overall, reviewed clinical trial articles demonstrated that the DPS was effective in detecting the medication adherence. Low detection in some investigations was attributed to participants who did not engage with the technology.^29^ For some participants, decreased engagement was due to local skin reactions from wearing the cutaneous patch (Proteus system).^27,29,35,39^

### User experience after real-world DPS use

Eleven of the 18 clinical trials assessed the user experience after participation in the study.^24,26,27,31,32,34,35,37–39,43^ Two pilot studies among these evaluated user experiences via semi-structured qualitative interviews at the end of the study period.^26,32^ Many participants agreed that the DPS was straightforward to learn and that these systems provided essential insights into their medication-taking behaviors. The majority additionally noted that the DPS was easy to operate on a daily basis and integrate into their existing routines.^27,31,32,34,35,37,38,43^ Regarding the digital pills themselves, participants reported that the over encapsulation of their medications with a digital pill did not change the palatability of the pills nor prevent them from ingesting their medications as directed.^26^ No studies reported DPS-related adverse events, except those using a cutaneous adherent patch as a receiver device^25,26^; in these studies, investigators reported cases of minor skin reactions from the adhesive, which was described as superficial dermal irritation or a self-resolving rash.^22,24,27–29,31,38,39,43^ In investigations that included supplemental reminder systems to help patients adhere to their medication while using the DPS, these reminder messages were found to be acceptable and drivers of sustained adherence.^32^

Several investigators also queried healthcare providers about their experiences interacting with DPS. Most reported that the systems provided them with greater confidence in adherence reporting compared to other electronic adherence monitoring systems such as the digital pillbox.^8^ DPS data also were found to be helpful for treating physicians and pharmacists to understand actual medication use. They reported that data from the DPS informed adherence recommendations given to participants.^31,34^ Notably, providers also reported concerns that DPS technologies, and the ancillary services required to operate them (e.g. smartphone, wireless Internet access, or phone data plans), may render them inaccessible to populations of lower socioeconomic status who may benefit most from use. Finally, providers suggested that a DPS could be implemented for brief periods of time as a booster approach for patients experiencing difficulties with adherence despite trying other, lower-intensity interventions.^32^

### Qualitative studies surrounding use of the DPS

Three qualitative studies that explored patients’ and providers’ acceptance of and willingness to use the DPS and integrate it into existing workflows were identified in this review. Study populations included substance-using men who have sex with men (MSM) who were taking pre-exposure prophylaxis (PrEP) for HIV prevention, individuals living with HIV, patients over age 65 years old who were taking cardiovascular medications, and clinicians and nurses involved in the care of breast cancer survivors. With respect to methodology, these studies utilized both individual semi-structured interviews as well as focus groups. Interview guides were designed by study investigators and grounded in behavioral theory. Investigations described the use of the Technology Acceptance Model, Consensual Qualitative Research Framework, Necessity Concerns Framework, and Perceptions and Practicalities Approach. These models were used to frame the interviews and focus group discussions around various aspects of the DPS encompassing patient and provider barriers and facilitators to using the digital pill as an adherence measurement system. They were also used to explore how a DPS may be integrated into existing clinical and individual workflows.^40–42^

In one study, semi-structured qualitative interviews were conducted with MSM using PrEP for HIV prevention, who were prospective DPS users. Most participants perceived the DPS to be an innovative and useful tool for measuring ingestions that has the potential to improve medication adherence. They largely expressed a willingness to use the DPS in theory, given their difficulty adhering to medications, including PrEP, when engaging in HIV risk behavior; however, the lived experiences of such participants following actual DPS use may differ. Many participants also had concerns about the large size of the wearable receiver and suggested that integrating it with a smart watchband, phone case, or smartphone itself would make the technology more acceptable.^40^

A second qualitative study involved a focus group discussion, conducted with patients over the age of 65 years old who were taking cardiovascular medication, to solicit feedback on using technology for improving the way they take medications. Participants reported that technology such as the DPS could prevent adverse effects before they occur and could therefore be more cost-effective than current systems for managing adherence. Participants also noted that the DPS could be particularly useful for individuals with cognitive impairments by making their lives less dependent on other people and by providing opportunities for instantaneous intervention when necessary.^41^

In the third qualitative study, semi-structured interviews were conducted with healthcare providers to explore perspectives around adherence to hormonal therapy among breast cancer survivors. Providers reported the primary reasons for nonadherence among their patients included medication side effects, medication costs, forgetting, a lack of knowledge on why adherence is important and conflicting information on how to take their medications. Providers were confident that a DPS would improve their ability to monitor their patients’ adherence, and that having access to accurate, instantaneous ingestion data could improve the doctor-patient relationship. Some reported concerns that implementation of a DPS could become a liability concern due to the limited time available for doctors to remain up to date on their patients’ adherence. Additionally, providers anticipated several barriers to implementing the DPS, including a general lack of accessibility for patients of low socioeconomic status, safety and privacy considerations, and the practicality of wearing and operating the receiver during ingestions. They reported that the DPS would likely deliver the most benefit for elderly patients.^42^

## Discussion

Obtaining instantaneous, direct confirmation of medication ingestions can help to facilitate continuous adherence to key medication regimens, while simultaneously detecting nascent lapses in adherence. The application of a DPS to measure medication adherence remains an attractive alternative to directly measuring medication ingestions at high resolution. Episodes of suboptimal adherence may occur in contexts where empirically supported behavioral interventions targeting adherence can be delivered to reduce nonadherence and reinforce medication-taking behaviors.^44^ This review demonstrates that the use of DPS is broadly feasible across multiple disease states where adherence is closely linked to significant morbidity and mortality. It also highlights key areas of research that are still needed prior to broad-scale clinical deployment of such systems.

Pilot demonstration projects describing the use of a DPS indicate feasibility across a wide range of disease states and pharmacotherapy, including opioids, solid organ transplant, HCV, HIV treatment, diabetes, hypertension, and tuberculosis therapy.^22,24–29,31–35,37–39,43^ Overall, these investigations demonstrated the feasibility and general acceptability of DPS operated in the real world by a diverse array of patients, and link pilot adherence data with certain biological endpoints. It is important to note, however, that the overall aims of many of the pilot studies were not to understand the impact of medication adherence on biological outcomes, but rather to understand the feasibility of using a DPS to measure adherence in order to more accurately assess the relationship between ingestion patterns and biological outcomes. These studies also described the operation of the DPS over periods of time ranging from one week to six months. The duration of DPS use in these investigations reflects the intervals between potential clinical visits relevant to these disease states and suggests that the integration of DPS into clinical adherence monitoring is feasible from a technological standpoint. Finally, several investigations also considered the use of multiple digital pills at once and demonstrated that the technology can be effectively utilized to measure adherence to several different medication regimens or regimens that require ingestion of two pills or dosage forms simultaneously.^22,26,29^

Several investigations in this review provided insight into how data from DPS could aid clinical care teams. Instantaneous ingestion data is perceived as valuable to pharmacists, care coordinators and physicians, as it delivers contextual information that can help them understand the potential etiologies for treatment failure (e.g. in the context of antihypertensives), or help them to make individualized recommendations for their patients.^31,34,37,43^ Qualitative studies have also demonstrated that healthcare providers are receptive to, and trusting of, data collected by DPS.^42^ Despite this, it remains unclear how best to integrate DPS data into the arc of clinical care, including who should be primarily responsible for receiving and monitoring patients’ adherence data, as well as how best to respond to nonadherence events detected by DPS. It is plausible that DPS data could be utilized at routine clinic visits in the same fashion as other measures of adherence. Informed by DPS ingestion data, specific strategies could then be delivered to patients to improve adherence, or, in the context of treatment failure, the data could be used to aid a clinical team in selecting alternative pharmacotherapy. Another possibility would be the development of algorithms that interpret instantaneous adherence data, and then automatically provide nudges to patients in the form of behavioral interventions, as well as notifications to clinicians in the event of suboptimal adherence or even impending nonadherence. These potential avenues represent gaps in the DPS literature that warrant exploration in future investigations.

While most users provided positive feedback surrounding their experience using the DPS, many suggested technical improvements to DPS hardware, specifically the wearable receiver portion of the system. Commercially available DPS currently require the use of an additional wearable device, either an adhesive cutaneous patch (Proteus Digital Health) or an off-body wearable device (etectRx) to collect the radiofrequency signal from the ingestible sensor once activated in the stomach, thereby logging an ingestion event. Until signal boosting improves to permit the capture of ingestible sensor data without a relay device, the operation of such receivers will continue to be a consideration when deploying DPS. Targeted training programs, or specific instruction around management of relay devices associated with the DPS, may help to mitigate these concerns and improve adherence to the technology.^26^

Finally, a significant amount of qualitative work has been conducted surrounding the application of DPS for various disease states.^41,42^ These investigations have identified common facilitators to uptake of DPS despite the heterogeneity of barriers to adherence across different diseases. Individuals are generally accepting of the DPS and perceive the technology as helpful for providing insights into adherence behavior and for receiving contextualized interventions to help correct for nonadherence.^40,41^ Qualitative research with individuals who utilized DPS in clinical trials has also demonstrated that, despite hardware-related barriers to use, there is a desire to adopt and continue use of DPS in their current iteration. Moreover, although some participants have raised concerns surrounding the privacy of DPS data, current work suggests that these considerations may not be an issue among patients who are actively using DPS for medication adherence.^32^ Further qualitative work may help to identify key barriers around DPS use, including data-related concerns, that can be mitigated to further improve the acceptability of the technology. Additionally, the concept of the Hawthorne Effect around DPS use remains unclear; qualitative work suggests that participants do not feel social pressure to become particularly adherent because of the instantaneous sensing nature of the DPS, yet many of the human clinical trials described in this review do not focus on potential confounding via the Hawthorne Effect.

Overall, this review demonstrates that while DPS remains nascent, there have been significant advances in understanding the feasibility and acceptability of such systems across high value diseases where adherence is paramount. The current literature suggests that DPS are accepted by patients in clinical trial settings and that DPS-generated adherence data is valuable to both patients and clinicians. Powered randomized controlled trials are still needed to understand the effects of DPS in improving adherence, both in terms of a quantitative comparison to other methods of adherence, and in the assessment of biological endpoints. Additionally, analyses of DPS cost effectiveness and clinical outcomes are lacking. This may be due to the wide variety of cost analyses dependent on a specific disease state, baseline adherence, and healthcare costs linked to complications associated with various diseases. In summary, DPS should be viewed as useful adherence monitoring tools for clinical trials and, potentially, in the clinical care setting.

### Limitations

This study had several limitations. First, we conducted a narrative review with stringent inclusion criteria; this review may have missed non-indexed articles, whitepapers, and other policy documents that consider DPS in the context of other adherence tools. Second, we excluded literature around technical development of signaling, energy harvesting, and hardware development. Third, we only included English language articles and may have therefore missed developments in DPS technology published in other languages.

## Conclusions

This narrative review demonstrates that DPS are a feasible and acceptable method for measuring medication adherence across a wide spectrum of disease states. In some instances, DPS may improve adherence and result in improved biological outcomes, suggesting that reinforced adherence from a DPS can mitigate certain outcomes associated with medication nonadherence. DPS users perceive on-demand access to their personal, instantaneous adherence data to be a significant facilitator for using a DPS, and clinicians viewed DPS adherence data as useful for informing discussions with patients regarding chronic disease management.

We anticipate that future refinements to the DPS technology will involve miniaturizing the existing wearable Reader device that is required to acquire the ingestible radiofrequency signal. While miniaturization may be viewed as a technological advancement, for some applications where target populations may lack access to smartphones, or in places where reliable cellular signals are unavailable, the existing system augmented by an integrated radio antenna within a wearable Reader device may be preferable. Given the current literature, we envision that DPS technology will be utilized for adherence measurement in emerging pharmaceutical trials, in efficacy studies of novel pharmacotherapy, and in the development of interventions linked to instantaneous adherence data and clinical use of DPS adherence data. Future research should be cognizant of the importance of demonstrating not only adherence patterns using the DPS, but its relationship with key biological outcomes in diseases studied.
